# A Jumbled Argument

**Published:** 1916-09-02

**Authors:** 


### "A JUMBLED ARGUMENT."

(THE "POOR-LAW OFFICERS' JOURNAL," AUGUST 18, 1916.)

The *Poor-Law Officers' Journal*, seeking to maintain the position of protector of Poor-Law officers' interests, shows a hypersensitiveness carried to the point of seeking for veiled insults.

In The Hospital of August 12 we published an article upon "Nurses and Central Examinations" in their relation to the future of British nursing. The gist of our argument was that, under the auspices of the College of Nursing, central examinations in nursing training will be held; that both hospital and Poor-Law infirmary nurses are prepared for this innovation; and that the standardisation of training which will follow will be for the benefit of nurses individually and of the nursing profession as a body. In our argument the *Poor-Law Officers' Journal* finds a prejudice against, and veiled insults to, Poor-Law nursing; these are purely imaginary! In fact, we agree, and know, that many Poor-Law trained nurses are "among the most proficient in the practice of nursing, and are in demand everywhere"; we have made no statement or innuendo to the contrary.

We did not state how the College of Nursing will carry out the details of the central examinations which we believe it will conduct because we do not yet know. The matter is one for the College, and one upon which, not unnaturally at this stage, no statement has been issued. We anticipate that arrangements may be made somewhat similar to those followed in connection with the examinations of the Central Midwives Board. We did not state that "central examinations are already in progress." To hold central examinations for nurses trained at Poor-Law infirmary training schools (and for those nurses alone) might differentiate Poor-Law nurses as a class apart, and they might even be excluded from such central examinations of wider scope as we had under consideration. Hence our expression of satisfaction that the Poor-Law institutions are still "free to present their nurses at central examinations side by side with those trained in the general hospitals." Our critic in the *Poor-Law Officers' Journal* says: "If a nurse comes up for examination . . . the sole point is whether she can satisfy the examiner in regard to her proficiency in her profession." Exactly; that is also one of our points; that a nurse, wherever trained, should attain a publicly recognised standard of qualification and register. The need of such a standard is shown by the fact that the Local Government Board recognises training under certain conditions, and has adopted the only "standard" at present available.

The "prejudice against the work of the Poor Law" exists only in the pages of The Hospital when those pages are read with a jaundiced eye. Such prejudice exists elsewhere, and the adoption of central examinations for nurses will be a step towards its removal.

